# Comparative evaluation of structured oil systems: Shellac oleogel, HPMC oleogel, and HIPE gel

**DOI:** 10.1002/ejlt.201400553

**Published:** 2015-05-05

**Authors:** Ashok R Patel, Koen Dewettinck

**Affiliations:** Faculty of Bioscience Engineering, Vandemoortele Centre for Lipid Science and Technology, Laboratory of Food Technology & Engineering, Ghent UniversityGhent, Belgium

**Keywords:** Edible applications, HPMC, Microstructure, Oil structuring, Rheology, Shellac

## Abstract

In lipid-based food products, fat crystals are used as building blocks for creating a crystalline network that can trap liquid oil into a 3D gel-like structure which in turn is responsible for the desirable mouth feel and texture properties of the food products. However, the recent ban on the use of trans-fat in the US, coupled with the increasing concerns about the negative health effects of saturated fat consumption, has resulted in an increased interest in the area of identifying alternative ways of structuring edible oils using non-fat-based building blocks. In this paper, we give a brief account of three alternative approaches where oil structuring was carried out using wax crystals (shellac), polymer strands (hydrophilic cellulose derivative), and emulsion droplets as structurants. These building blocks resulted in three different types of oleogels that showed distinct rheological properties and temperature functionalities. The three approaches are compared in terms of the preparation process (ease of processing), properties of the formed systems (microstructure, rheological gel strength, temperature response, effect of water incorporation, and thixotropic recovery), functionality, and associated limitations of the structured systems. The comparative evaluation is made such that the new researchers starting their work in the area of oil structuring can use this discussion as a general guideline.

**Practical applications:**

Various aspects of oil binding for three different building blocks were studied in this work. The practical significance of this study includes (i) information on the preparation process and the concentrations of structuring agents required for efficient gelation and (ii) information on the behavior of oleogels to temperature, applied shear, and presence of water. This information can be very useful for selecting the type of structuring agents keeping the final applications in mind. For detailed information on the actual edible applications (bakery, chocolate, and spreads) which are based on the oleogel systems described in this manuscript, the readers are advised to refer our recent papers published elsewhere. (Food & Function 2014, 5, 645–652 and Food & Function 2014, 5, 2833–2841).

## Introduction

**Figure 1:**
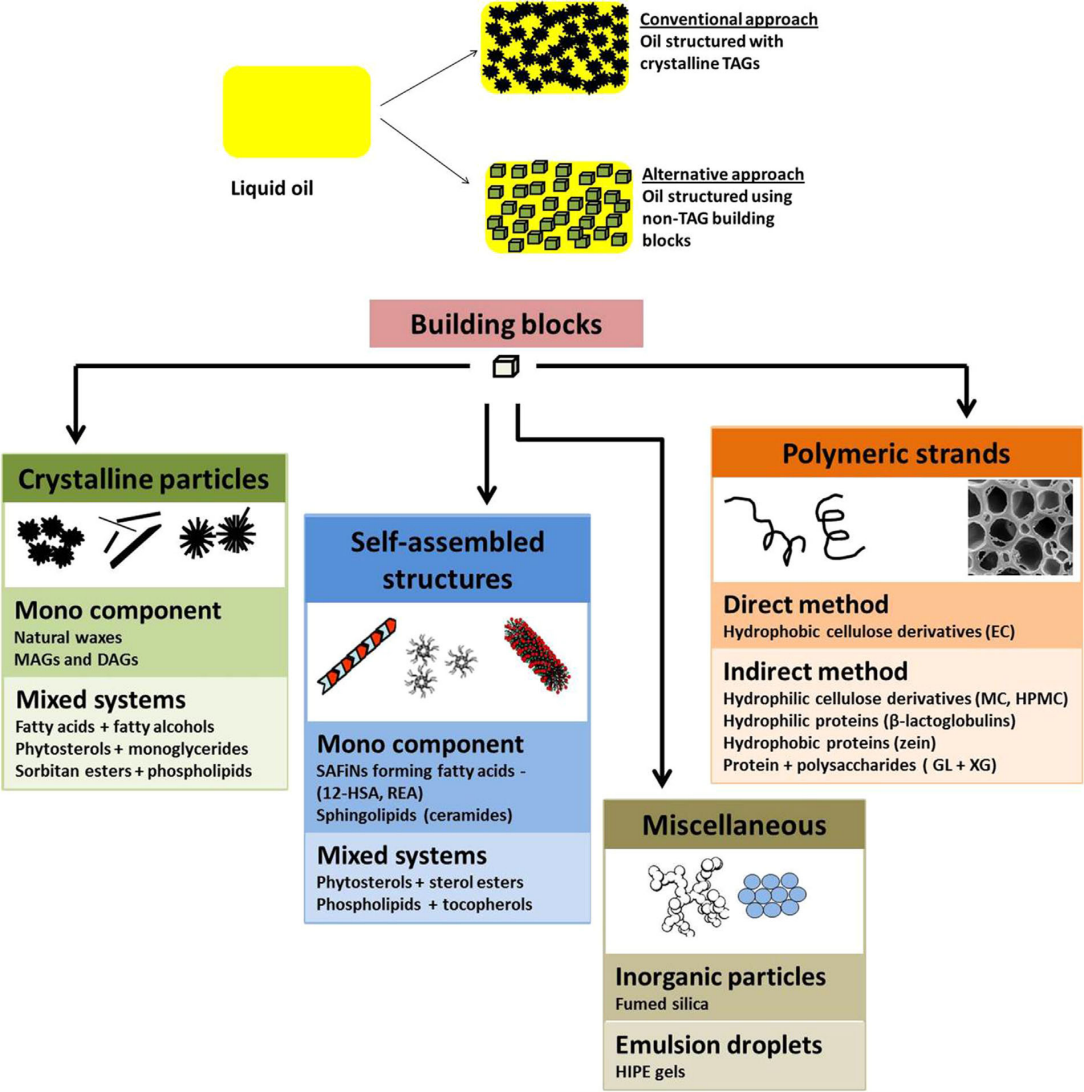
Schematic representation of oil structuring through conventional approach using crystalline TAGs as building blocks and alternative approach using non-TAG building blocks. Building blocks are categorized into different classes with suitable examples.

The functionality and desirable texture properties of commonly consumed lipid-based food products are governed by the underlying colloidal network of fat crystals that is responsible for trapping liquid oil into a 3D “gel-like” structure [1]. The oil structuring in this case is caused due to the limited solubility of high melting TAGs in the oil, upon cooling a hot solution, the TAG molecules crystallize out of the liquid broth forming crystals that interact together to form a network [2,3]. This conventional way of oil structuring relying on the use of fat crystals or crystalline TAG molecules as building blocks suffers from two basic shortcomings. Firstly, to achieve efficient oil structuring, usually a high fraction of crystalline TAG phase (≥20%) is required and secondly (and more importantly), the crystalline TAG molecules are rich in saturated and or trans fatty acids and excessive consumption of these unhealthy fats is linked to cardiovascular disorders [2,4–7]. Hence, a lot of effort has been put recently into exploring alternative ways of structuring oils by identifying newer building blocks that are capable of structuring oils at much lower volume fractions in order to generate gels with higher concentration of liquid oils (>90 wt%) [2,7–10]. In the last few years, a number of gelator molecules have been researched for edible oil structuring (see Fig. 1), the basic building blocks (supramolecular assemblies) formed by these molecules fall into one of the following categories: (i) crystalline particles; (ii) self-assembled structures of low molecular weight compounds (fibers, strands, tubules, reverse micelles, mesophases, etc.); (iii) self-assembled structures of polymers or polymeric strands; and (iv) miscellaneous structures like colloidal particles and emulsion droplets. The building blocks can be formed by single component or mixture of components (mixed systems) and the formation of building blocks could be achieved through direct method (usually by dispersing gelator molecules in oil medium at high temperatures followed by cooling) or indirect method in case of hydrophilic polymers where dried microstructures are created by stripping off the water from hydrated polymer solutions [2,7,8,11–13].

The current paper gives a brief account of three different structured systems: wax-based oleogel, polymer oleogel, and high internal phase emulsion gels prepared with shellac wax, HPMC, and water droplets (gelled using LBG:Car) as building blocks, respectively. These systems were developed in our laboratory and have been previously communicated externally. The aim of the current paper is to specifically discuss the comparative differences in terms of the preparation process (ease of processing), properties of the formed systems (microstructure, rheological gel strength, temperature response, effect of water incorporation, and thixotropic recovery), functionality, and associated limitations of the structured systems. The comparative evaluation is made such that the new researchers starting their work in the area of oil structuring can use this discussion as a general guideline.

## Materials and methods

### Materials

Shellac wax, SSB® Cera 2 (acid value: 2–25 mgKOH/g and saponification value: 40–60 mgKOH/g) was received as a generous gift sample from SSB Stroever GmbH & Co. KG., Germany. Different viscosity grades of HPMC (AnyAddy, 15, 50, 100, and 4000 cps, Samsung Fine Chemicals) were received as gift samples from Harke FoodTech, Germany. LBG and Car were provided by Cargill R&D (Vilvoorde, Belgium). RPO, SFO, PGPR, Palsgaard 6111® (fully hydrogenated rapeseed oil with high content of erucic acid) a commercial crystal starter and commercial shortening were received as gift sample from Vandemoortele Lipids N.V., Belgium. Sudan red and Rhodamine B were purchased from Sigma–Aldrich Inc., USA. Water purified by the MilliQ system was used for all the experiments.

### Preparation of structured oil systems

#### Shellac oleogels

Shellac wax was accurately weighed and dispersed in RPO to achieve a concentration range of 0–6 wt%. The dispersions were heated at 90°C for 30 min under mild agitation (200 rpm) using magnetic stirrer (Model EM3300T, Labotech Inc., Germany). The clear oily dispersions were then cooled to room temperature resulting in the formation of shellac oleogels. Further, water-in-oil emulsions were prepared by first mixing the heated oleogel sample and water at 90°C under continuous stirring (at 11 000 rpm) using a high energy dispersing unit (Ultraturrax®, IKA®-Werke GmbH & Co. KG, Germany) followed by cooling the mixture to room temperature.

#### HPMC oleogels

The preparation of oleogels included following steps:

- Aqueous foam preparation and drying: Accurately weighed samples of HPMC were dissolved in water to achieve 2 wt% polymer solutions. These solutions of different viscosity grade HPMC were then aerated with the use of Ultraturrax®. The aqueous foams were then frozen at −23°C overnight before subjecting them to freeze drying using VaCo5 lyophilizer (ZirBus Technology, Germany) to obtain porous cryogels.
- Oleogel preparation: Different quantities of porous cryogel were weighed and submerged into SFO and left overnight for oil sorption. The oil-sorbed cryogels were then sheared at 11 000 rpm using Ultraturrax® to get the oleogels which had varying amount of polymer weight (1–5 wt%).

#### High internal phase emulsion (HIPE) gels

Oil and water phases were prepared beforehand by dispersing PGPR (at 0.4 wt% of total emulsion) in SFO and LBG and Car in different proportions in water, respectively. Water in oil emulsions (*ϕ*_water_ = 0.75) were prepared by shearing the heated mixture of oil and water phases (70°C) using Ultraturrax® (at 11 000 rpm) followed by cooling to room temperature resulting in gelled emulsions.

### Characterization of structured oil systems

#### Microstructure studies

The microstructure was studied using Leica DM2500 microscope (Leica Microsystems, Belgium) under normal and polarized light. For confocal microscopy, samples were imaged using a Nikon A1R confocal microscope (Nikon Instruments Inc., USA). Excitation was performed by means of a 488 nm Ar laser and fluorescence was detected through a 525/50 bandpass filter. Images were acquired and processed with Nikon NIS Elements software. For cryo-SEM, samples were placed in the slots of a stub, plunge-frozen in liquid nitrogen, and transferred into the cryo-preparation chamber (PP3010T Cryo-SEM Preparation System, Quorum Technologies, UK) where it was freeze-fractured and subsequently sputter-coated with Pt and examined in JEOL JSM 7100F SEM (JEOL Ltd., Tokyo, Japan). For water containing samples, sublimation step was included to get rid of water.

#### Rheological measurements

The rheological measurements were carried out on advanced rheometer AR 2000ex (TA Instruments, USA) using a parallel plate geometry of diameter 40 mm. A range of measurements including flow tests and oscillatory measurements like amplitude (strain and stress), frequency, temperature, and time sweeps were performed. Except for temperature ramps, the measurements were done at a constant temperature of 5°C for shellac oleogels and HIPE gels and 20°C for HPMC oleogels. Amplitude strain or stress sweeps were carried out at a frequency of 0.25 Hz for shellac and HPMC oleogels and 1 Hz for HIPE gels. Other oscillatory tests were done at a constant strain or stress values under LVR. In addition, a 3 interval thixotropy test (3-ITT) was conducted on the shellac oleogels and HPMC oleogels where the samples were subjected to alternative cycles of low and high shear rates (0.1 and 10 s^−1^, respectively) and the results are shown as viscosity versus time plots.

#### Thermal analysis

For shellac oleogels, the thermal parameters were studied using a Q1000 differential scanning calorimeter (TA Instruments, USA) on samples weighing 10 mg in flat-bottomed aluminum pans. The samples were subjected to heating and cooling cycles from 5 to 100°C and back at a constant cooling rate of 1°C/min. The thermal parameters were obtained from the heat flow curves with the help of TA Universal Analysis software.

#### Droplet size analysis of emulsions

Water droplet size analysis of the w/o emulsions was performed by pulsed field gradient Nuclear Magnetic Resonance (pfg-NMR) on a benchtop Maran Ultra spectrometer (Oxford Instruments, UK) operating at a frequency of 23.4 MHz in combination with the droplet size application (Resonance Instruments Ltd.). Samples were analyzed at 5°C to minimize inter-droplet water diffusion. To suppress the NMR contribution of the fat phase, pfg-NMR experiments were conducted using an inversion recovery-stimulated echo pulse sequence. In the performed experiments, the diffusion time (Δ) was set to 0.2 s, the gradient strength was fixed at 1.74 T/m, while the gradient duration (δ) was varied in 17 steps from 400 to 4500 ms. By measuring the echo attenuation ratio of the NMR signal as a function of the gradient duration, it is possible to determine the hindered diffusion behavior and hence, the droplet size distribution.

#### Characterization of cake batters

In order to evaluate the functionality, cake batters were prepared using the three-structured systems as fat phases. Classic 4/4 sponge cakes were prepared using 300 g wheat flour, 13 g baking powder, 300 g liquid whole egg, 300 g sugar, and 300 g fat phase. The cake batter was prepared by mixing these ingredients in Kitchen Aid® mixer followed by baking at 175°C for 35 min. For comparison, commercial shortening and liquid sunflower oil were used positive and negative references, respectively. The cake batters were compared in terms of air incorporation and consistency using density and oscillatory rheological measurements. For density measurements, batter samples filled in a glass cylinders with known volume were accurately weighed in triplicates. For rheology studies, amplitude sweeps (strain = 0.1–100%) were conducted at 20°C using a parallel plate geometry.

## Results and discussion

### Shellac oleogels

#### Formation of shellac oleogels

The DSC curve of shellac wax shown in Fig. S1a is characteristic of a wax as indicated from multiple melting peaks (and corresponding crystallization peaks) that are representative of different components. Shellac wax is purified from the secretion of lac insect, *Laccifer lacca*. It is widely used material in food and pharmaceutical fields as coating, glazing, and film forming agent [14,15]. Chemically, it is composed of a complex mixture of polar and non-polar components consisting of long chain fatty acids, wax esters, fatty alcohols, and alkanes [16].

**Figure 2:**
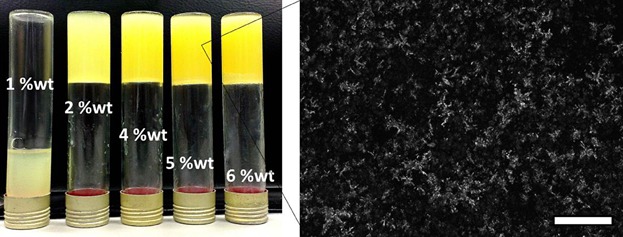
Photograph of oleogels prepared at varying concentrations of shellac wax in rapeseed oil, the polarized light microscopy image of oleogel with shellac 5 wt% (scale bar = 200 μm).

**Figure 3:**
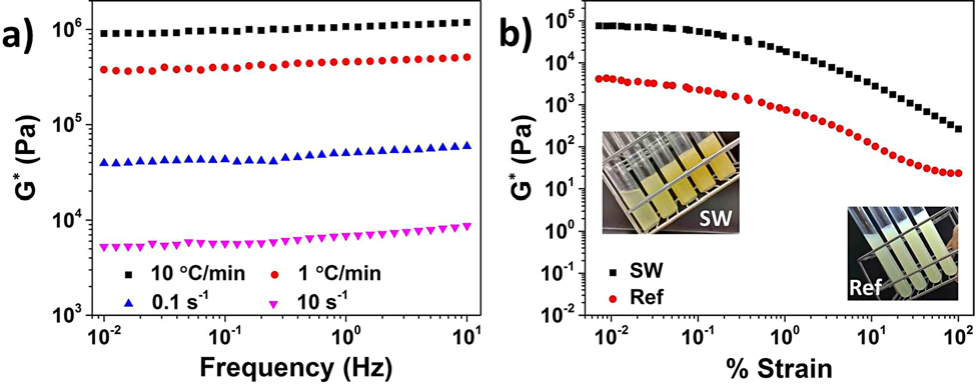
(a) Frequency sweeps (percent strain = 0.01) of oleogels prepared at 5 wt% under low and high cooling as well as shear rates and (b) amplitude strain sweep of shellac oleogel (prepared at 5 wt%) compared to reference oleogel prepared using commercial crystal starter, Palgaard 6111® (at 10 wt%). Insets: photographs of a series of shellac 1, 2, 4, 5, and 6 wt% and reference oleogels (4, 6, 8, and 10 wt%).

The gelation of oil by shellac wax was a result of a crystalline network formation (Fig. 2) with a minimum gelling concentration of 2 wt%. Moreover, the cooling curves of oleogels prepared at different concentrations (Fig. S1b) shows that at higher concentration, multiple crystallization peaks start appearing which is further indicative of the crystallization of multi components of the wax [17]. Since the gelation is a result of crystallization, the effect of factors such as cooling rates and shearing rates on oleogel formation was studied using oscillatory rheological measurements (frequency sweeps, as shown in Fig. 3a)

As seen from the figure, the oleogels prepared at higher cooling and lower shearing rates, respectively, showed better rheological profiles as indicated by relatively higher G* values (which is a measure of the total resistance of a system towards deformation) and lower frequency dependence of the modulus. The effect of cooling rate was as expected because a higher cooling rate results in the formation of a large number of smaller crystals which in turn leads to stronger crystal–crystal interactions and consequent network formation [18–21]. The microscopy images provided in Fig. S2 clearly shows a denser crystal network of the sample prepared at a higher cooling rate. The relatively better consistency of sample prepared at lower shear rate suggests that the weak association of crystals into a network (probably driven by weak forces such as London Dispersion force) is sensitive to shear.

#### Properties of shellac oleogels

The oil structuring properties of shellac wax was compared to the reference Palsgaard® 6111 (which is a commercial crystal starter used for oil structuring). It was found that a much higher concentration of latter was required for oil gelation with minimum gelling concentration of 8 wt% for Palsgaard® 6111 compared to just 2 wt% for shellac (Fig. 3b inset). Moreover, the difference in the value of G* for 5 wt% shellac and 10 wt% Palsgaard 6111® oleogel by almost a decade over entire strain range (Fig. 3), indicates that shellac oleogels had comparatively higher gel strength. The higher strength of shellac oleogels (at a significantly lower concentration) could be attributed to the self-assembling properties of shellac which triggers a higher crystal–crystal interactions leading to a stronger network [22].

**Figure 4:**
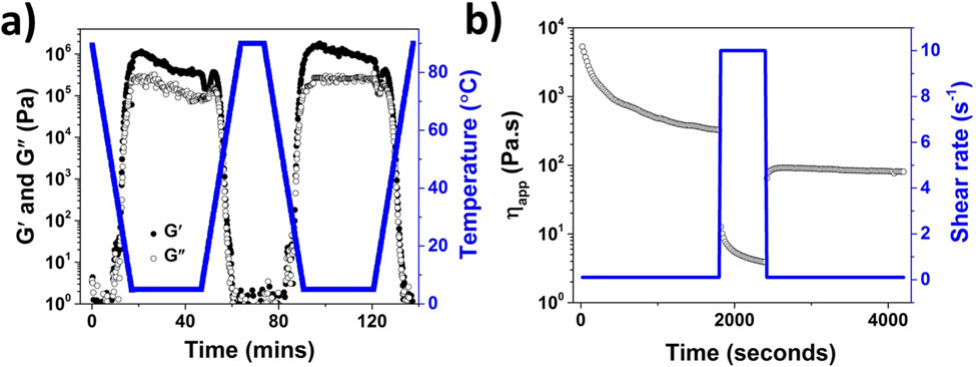
(a) *G*′ and *G*″ of shellac oleogel measured over alternating cooling and heating cycles and (b) viscosity plot from 3-ITT of shellac oleogel.

In addition to structuring efficiency (gelling liquid oils at low crystalline volume fractions), the potential use of oleogels to substitute for fat functionality in food products will also be dictated by other properties of formed oleogels such as reversible microstructure development as a function of temperature and shear history as well as the possibility of structure stability in the presence of water [23]. Thermo reversibility (reversible transformation from liquid to gel as a function of temperature) and thixotropic recovery (structure recovery after shear removal) were studied using rheological measurements and the effect of the presence of water on the microstructure was studied by formulating w/o emulsions using oleogels as the oil phase. The *G*′ and *G*″ measured over the cooling and heating cycles (Fig. 4a) confirms that shellac oleogel shows reversible gel to sol transformation as a function of temperature. This interesting property could be beneficial when considering edible applications where a thermo reversible behavior is desired for organoleptic properties (such as mouth feel) of fat-based products. The structure recovery properties were evaluated by studying 3-ITT where the viscosity changes were followed as a function of time under alternative intervals of low and high shear rates (Fig. 4b). Ideally, a sample is considered to have a good thixotropic recovery if the peak viscosity value in the third interval is at least 70% of the viscosity value obtained at the end of first interval. From Fig. 4b, it can be seen that shellac oleogel showed only a partial structure recovery (percent recovery <30%) and thus, the suitability of shellac oleogel can be considered to have some limitations in applications where a reversible structure breakdown and recovery is desired.

**Figure 5:**
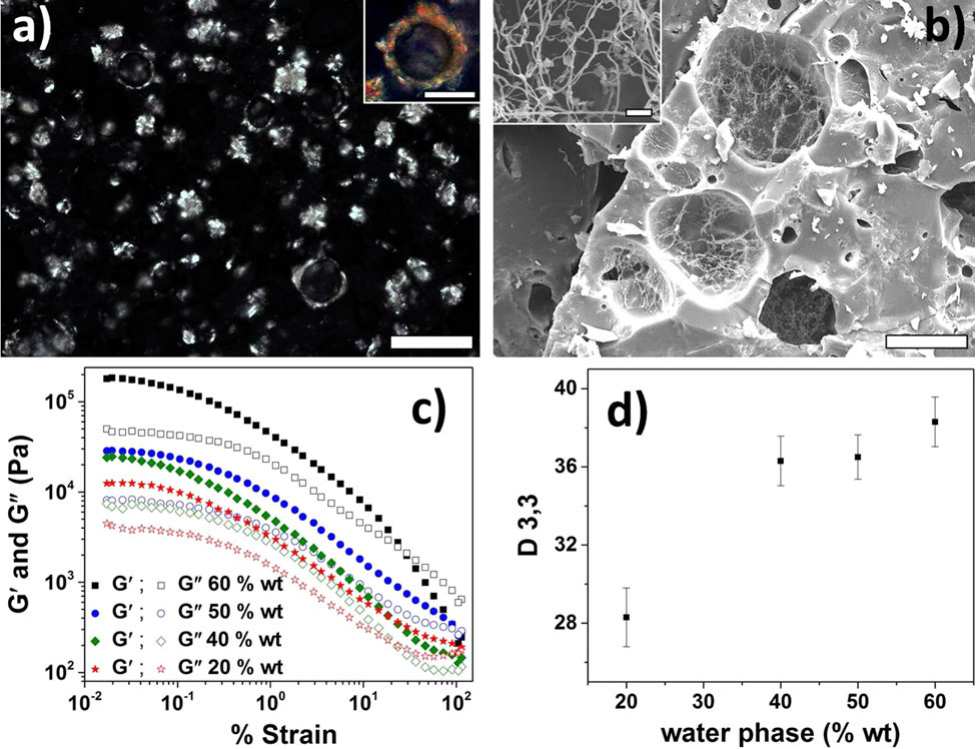
(a) PLM image of emulsion showing water droplets and wax crystals (scale bar = 50 μm), inset: magnified image of water droplet showing the presence of crystal at droplet interface (scale bar = 25 μm); (b) cryo-SEM image of freeze-fractured emulsion sample (scale bar = 25 μm), inset: magnified image of the network of fine crystallites left after the removal of dispersed water through sublimation (scale bar = 200 nm); (c and d) comparative rheology and mean droplet sizes, respectively, of emulsion formed at water phase levels of 20–60 wt%).

To evaluate the effect of water, oleogels were used as oil phase to prepare w/o emulsions by cooling a mixture of melted oleogel and water under continuous shearing. The initial dispersion of water droplets could be attributed to the surface active fatty alcohols present in shellac [24]. With further drop in temperature, crystallization occurs in bulk as well as at the water–oil interfaces. PLM and Cryo-SEM images of emulsion is shown in Fig. 5. As seen from Fig. 5a, the stabilization of emulsion was a result of bulk as well as interfacial crystallization. The presence of crystallites at the interface is further confirmed from the cryo-SEM image of freeze fracture sample of emulsion where the water is removed through sublimation (Fig. 5b). To evaluate the possibility of using shellac oleogels for low fat spread applications, emulsions with higher water contents (up to 60 wt%) were prepared. The comparative rheology and mean droplet size results of these emulsions are shown in Fig. 5c and d. As expected, the increase in the water content led to an increase in both the consistency and the mean size of dispersed droplets in the emulsions. The increase in the consistency of emulsions with increased water incorporation indicates that the increased interfaces contributes to the overall rheology of the emulsions. The possibility of creating such stable emulsions without any added emulsifiers suggests a strong emulsify role of the polar components inherently present in shellac wax as well as the Pickering stabilization provided by interfacial accumulation of fine crystallites of shellac wax.

### HPMC oleogels

#### Formation of HPMC oleogels

Among various structuring agents explored for oleogelation, polymers appear to be the most promising ones because there are many polymers that are approved for use in foods and most of them have been well characterized. However, since most of the food polymers are inherently hydrophilic in nature, they are ineffective in structuring oils due to their limited dispersibility [25]. Ethyl cellulose, EC (a hydrophobic cellulose derivative) is the only known polymer to gel edible oil through direct dispersion of polymer in oil. However, the process used for dispersing EC requires heating of the polymer dispersion in oil to a temperature higher than the glass transition temperature of EC (130–140°C) [26].

**Figure 6:**
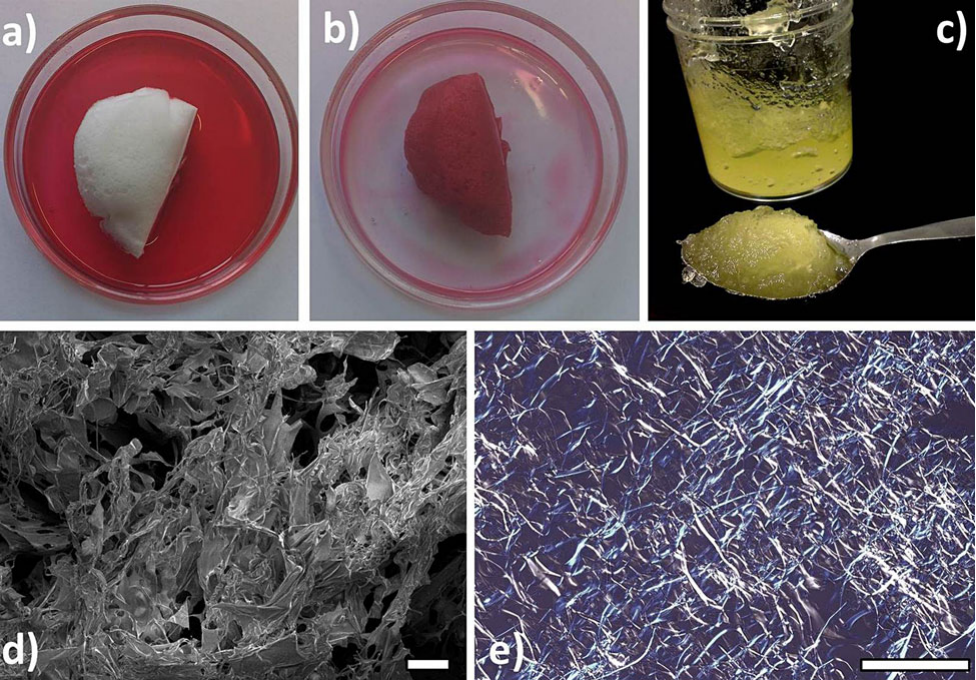
(a and b) Porous cryogel absorbing over 100 times its weight of liquid sunflower oil doped with Sudan red; (c) oleogel containing 98 wt% liquid sunflower oil prepared by shearing oil-sorbed cryogel; (d) cryo-SEM image of dried cryogel showing open porous structure (scale bar = 100 μm); and (e) PLM image of oleogel showing a network of birefringent polymer strands (scale bar = 200 μm).

Functionality of polymers to form structural framework in aqueous solvent is attributed to their hydration into an extended and open conformation which result in stronger molecular interactions [27,28]. Some food polymers such as proteins and modified polysaccharides are surface active and conformational framework can be created from water dispersions of these polymers by first promoting their adsorption to oil–water interfaces followed by removal of water as demonstrated by us previously [29,30]. Similarly, water-stripped dried microstructures can also be created from water dispersions of hydrophilic polymers by using colloid with air–water interfaces as templates [30]. Specifically, to prepare HPMC oleogel, water solution of HPMC was first aerated to generate aqueous foam which was subjected to freeze drying in order to obtain a porous cryogel. The porous cryogel showed excellent oil absorption properties, absorbing liquid sunflower oil at more than 100 times its own weight (Fig. 6a and b). This oil-sorbed structure was further sheared to obtain viscoelastic oil gels at liquid oil content of 95–98 wt%. The microstructure of dried cryogel (Fig. 6d) was akin to a reticulated solid foam with open cell structure which is responsible for high oil absorption capacity [31]. The microstructure of sheared oleogel was also studied using PLM. Interestingly, as seen from Fig. 6e, the well-formed polymer network (responsible for trapping oil into a viscoelastic gel structure) was highly birefringent. There could be two possible explanation for this behavior, firstly, the birefringence arises due to the inherent semi-crystalline nature of cellulose derivatives and secondly, this could be a result of structural birefringence induced due to the alignment of polymer molecules at the air–water interface under freezing and subsequent drying through sublimation [32].

#### Properties of HPMC oleogels

**Figure 7:**
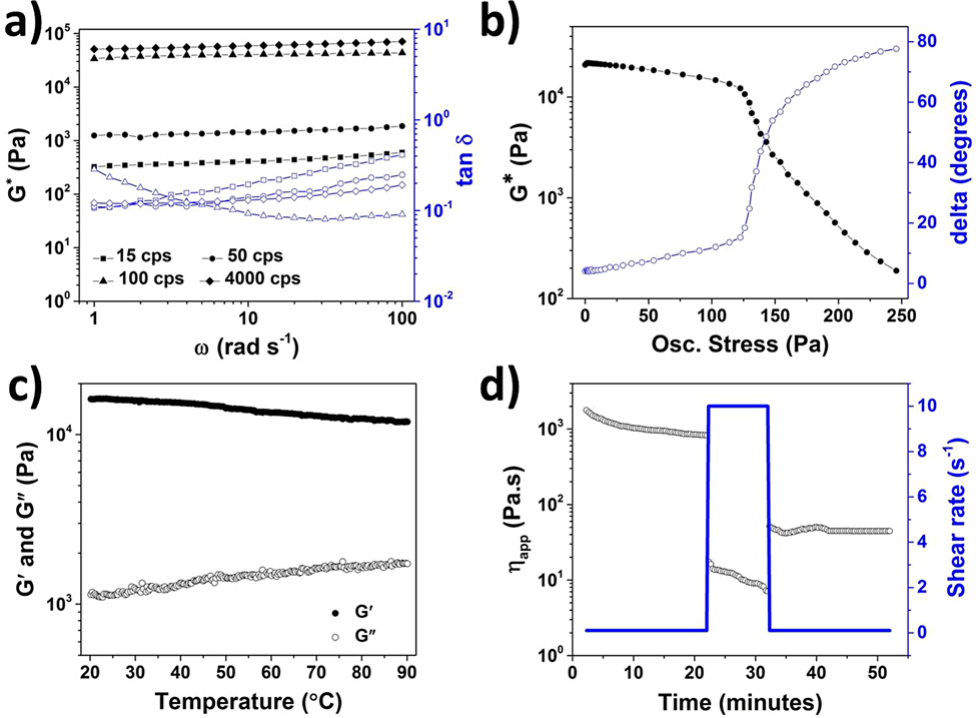
(a) Comparative frequency sweeps done on oleogel samples prepared using 2 wt% of HPMC 15, 50, 100, and 4000 cps; (b) amplitude (stress) sweep done on oleogel sample prepared using 2 wt% of HPMC 4000 cps; (c) temperature ramp for oleogel sample prepared using 2 wt% HPMC 4000 cps; and (d) viscosity plot from 3-ITT of oleogel prepared at 2 wt% of HPMC 4000 cps.

Cellulose derivatives like HPMC are synthetically prepared by substituting hydroxyl groups on cellulose backbone. Depending on the degree of polymerization (DP), different viscosity grades of HPMC are available. In the current work, four different viscosity grades (15, 50, 100, and 4000 cps) were screened. Comparative frequency curves of oleogels prepared using different viscosity grades of HPMC are showed in Fig. 7a. While, all the samples showed “gel-like” consistency (tan δ < 1), the gel strength of oleogels increased from HPMC 15 to 4000 cps. Hence, HPMC 4000 cps was used for all further investigations. The G* plotted against oscillatory stress (Fig. 7b) shows a narrow LVR and a prominent gel-sol transformation (delta degrees > 45°) at stress value close to 130 Pa. These results suggests that the gel is held together by weak internal forces which are overcome by higher applied stress.

Aqueous solution of cellulose derivatives like HPMC has a unique property of showing gelation at high temperature owing to an increased associative interactions among polymer chains at higher temperatures [33]. However, the oleogel samples showed a slight decrease in the gel strength when subjected to increasing temperatures (Fig. 7c). The “gel-like” consistency was still maintained at higher temperature with *G*′ higher than *G*″ over the entire temperature range and the difference between *G*′ and *G*″ being more than almost a decade. This difference in the temperature behavior of oleogel compared to aqueous solution is understandable since, the hydrophobic oil solvent minimizes the hydrophobe–hydrophobe associative interactions at higher temperatures [34]. In fact, the slight weakening of the gel strength at high temperatures suggests involvement of hydrogen bonding among polymer molecules forming the loose network that traps the liquid oil. The oleogel sample was also studied for thixotropic recovery properties (Fig. 7d). In comparison to shellac oleogels, the HPMC oleogel showed a much lower structure recovery in the third interval with values close to 5% compared to ≈30% for shellac oleogel. In addition, we also observed that the incorporation of water in these oleogels resulted in a complete structure loss due to the precipitation of aggregated polymer. Thus, in comparison to shellac-based oleogels, the applicability of HPMC oleogels is more limited and better suited for processes where oil leakage needs to be avoided at high temperatures such as baking.

### HIPE gels

#### Formation of HIPE gels

**Figure 8:**
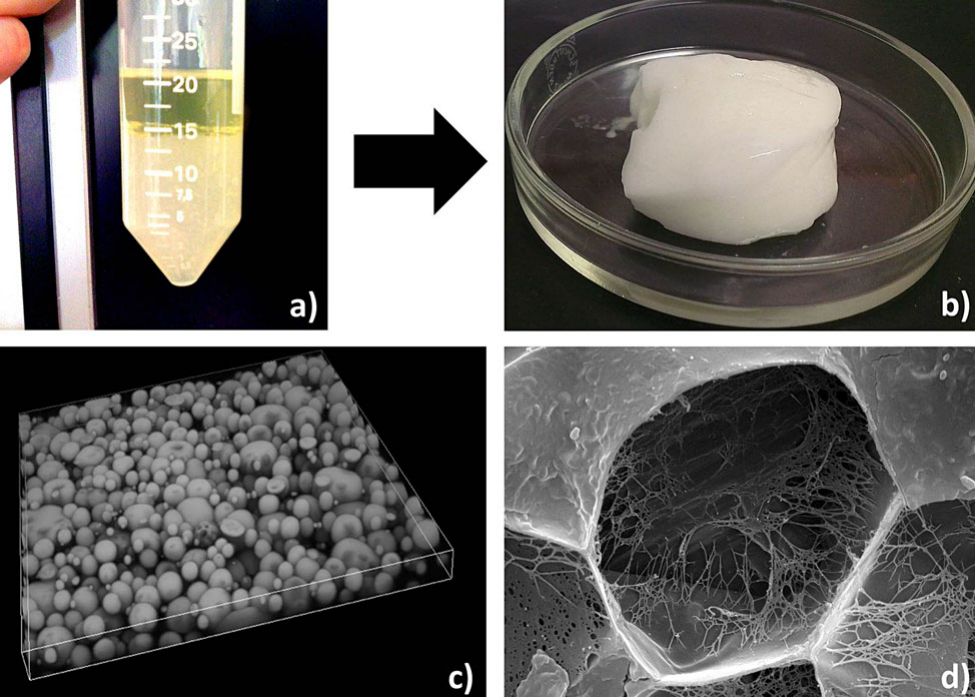
(a and b) The preparation of HIPE-gel by a high temperature emulsification of a mixture of oil and water (containing hydrocolloids) in presence of PGPR followed by cooling to room temperature; (c) 3D volume view created using stacked images obtained from CSLM (dimensions, *x* = 212 µm, *y* = 212 µm, and *z* = 15 µm); and (d) cryo-SEM image (image width = 15 µm) of freeze fractured sample after water phase was removed using sublimation.

HIPEs are defined as concentrated emulsion systems where the volume fraction of dispersed droplet phase is above 0.74, resulting in deformed dispersed droplet phase pack closely together separated with a thin film of continuous phase [35]. We used HIPEs as templates to generate oil continuous gels using low temperature triggered gelation of closely packed water droplets [36]. The preparation process for HIPE gel is shown in Fig. 8a and b. The gelling of water droplets was achieved through a combination of food-grade polymers (LBG and Car). The gelation of closely packed water droplets provides a structural framework (Fig. 8c) that supports the oil continuous phase, resulting in the formation of a self-standing gel. Further, the polyhedral microstructure of dispersed phase droplets can be clearly seen in the cryo-SEM image of freeze-fractured sample of HIPE gel (Fig. 8d). The internal microstructure of the droplet phase also shows a polymeric framework left behind after sublimation of water.

The emulsion gels were prepared at a constant water volume fraction (*ϕ*_water_) of 0.75 and stabilized using PGPR at a concentration of 0.4 wt% on total emulsion. The water phase was structured using LBG:Car at a total polymer concentration of 1 wt% (at LBG:Car ratio of 1:1). It is important to note that the emulsion prepared using ungelled water phase showed phase separation over 6 days of storage, thus, confirming that the droplet gelation is important for physical stabilization of the system.

#### Properties of HIPE gels

**Figure 9:**
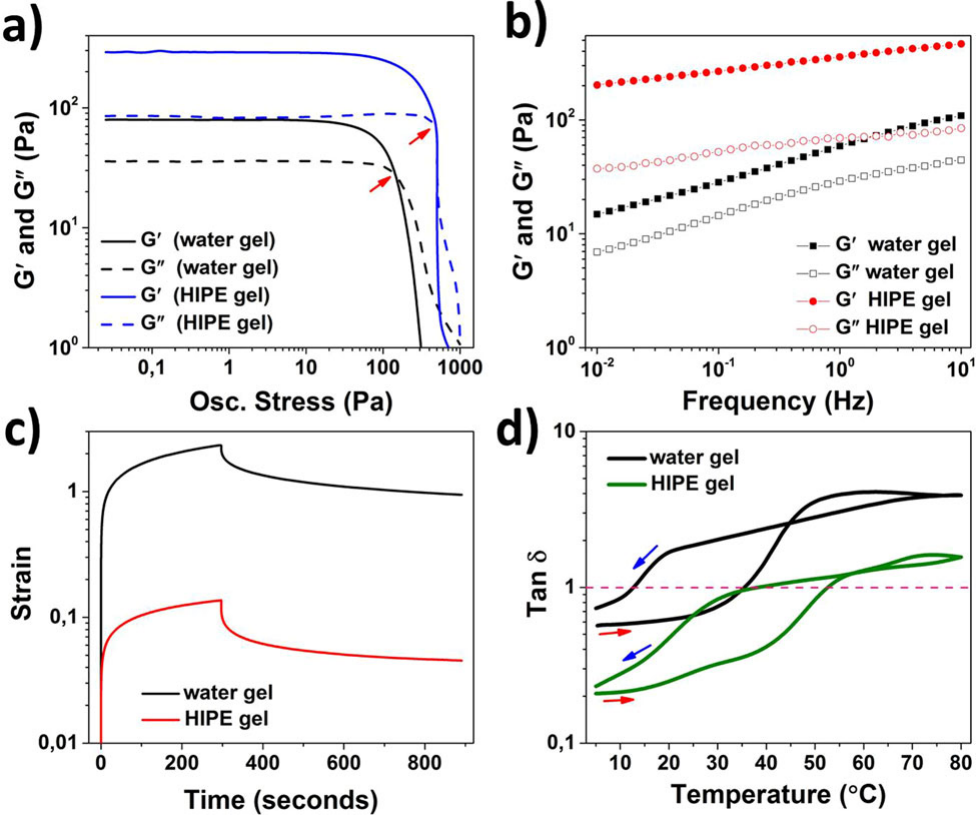
(a) *G*′ and *G*″ plotted against oscillatory stress (frequency = 1 Hz) for water gel and HIPE gel, oscillatory yield point or cross over point is indicated by red arrows; (b) comparative frequency sweep curves (stress = 1 Pa) for water gel and HIPE gel; (c) creep recovery curves (stress = 5 Pa) for water gel and HIPE gel; and (d) tan δ versus temperature for heating and cooling steps indicated with red and blue arrows, respectively, (stress = 1 Pa and frequency = 1 Hz).

The rheological properties of HIPE gel were studied and compared with the water gel using oscillatory and steady-state measurements. The amplitude and frequency sweeps shown in Fig. 9a and b, indicate that both the samples had a solid-like viscoelastic properties with *G*′ being higher than *G*″ in the LVR as well as throughout the entire range of applied frequency. In addition, relatively higher gel strength of HIPE gel over water gel is clearly confirmed from comparatively higher moduli values in the LVR; higher oscillatory yield stress (indicated by red arrows) and a lower frequency dependence of the moduli [37–39].

The viscoelastic properties of samples were also compared using creep recovery test. When viscoelastic samples are subjected to an instantaneous stress, the strain increases over time (a phenomenon known as creep) and the subsequent removal of the stress leads to a decrease in the strain (recovery) which, depending on the material properties, may or may not return to the zero strain over time [40,41]. On comparing the maximum creep (peak strain at the end of creep step) and the equilibrium strain (strain at the end of recovery step) (Fig. 9c), a comparatively softer structure of water gel can be confirmed from a relatively higher peak creep and equilibrium strain values as compared to HIPE gel. This results are in agreement with the results from oscillatory measurements shown in Fig. 9a and b. The reversible gel-sol transformation of samples can be studied by following the values of tan δ (*G*′*/G*″) plotted as a function of temperature for both heating and cooling steps (Fig. 9d). Both the water and the HIPE gel showed this reversible transformation, however, the critical temperature for gel-sol and sol-gel transformations (points where the curves crosses tan δ value of unity during the heating and cooling steps, respectively) were higher for HIPE gel relative to the water gel. It is important to note that the mean droplet size (<10 µm) of HIPE gel showed no changes when measured before and after the temperature treatment, suggesting an absence of droplet coalescence.

### Comparison of the functionality of the three-structured systems

The functionality of the fat phases to shorten the structure of cake batter and to stabilize air bubbles were compared using oscillatory rheology and density measurements. The calculated density values were 0.84, 1.08, 0.85, 0.93, and 0.94 g/mL for batters made using shortening, oil, shellac oleogel, HPMC oleogel, and HIPE gel, respectively. As expected, oil batter had the highest density indicating a low air incorporation due to the runny texture of the batter. On the other hand, batter made with shortening as fat phase showed a relatively lower density value (i.e., higher air incorporation) which further results in a much softer baked cake. The density values of batter made with the structured oil systems were significantly lower than oil batter suggesting that the structured consistency plays a role in the physical stabilization of air bubbles. Among the three systems, shellac oleogel showed highest air incorporation which could be attributed to the colloidal stabilization of air bubbles by surface active polar component (fatty alcohols) of shellac.

**Figure 10:**
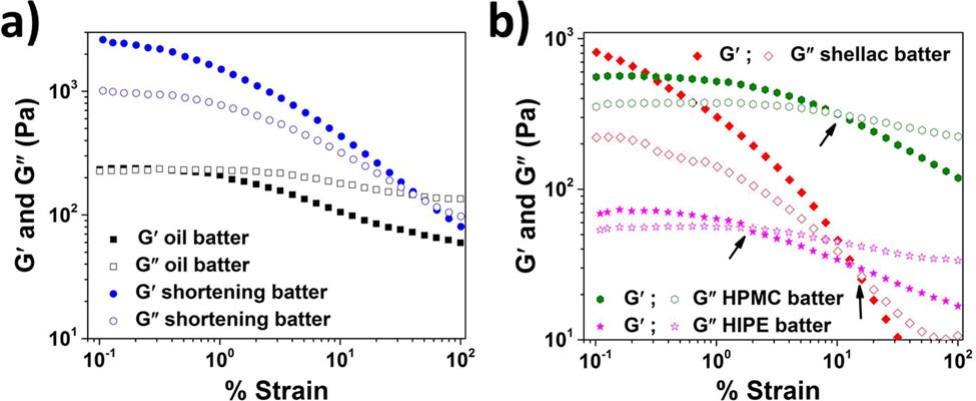
(a) *G*′ and *G*″ plotted as a function of percent strain (frequency = 1 Hz) for cake batters made using shortening and liquid oil as fat phases and (b) *G*′ and *G*″ plotted as a function of percent strain (frequency = 1 Hz) for cake batters made using 5 wt% shellac oleogel, 2 wt% HPMC oleogel and HIPE gel as fat phases. The crossover points of batters are indicated with arrows.

The runny and shortened consistency of batters made with oil and shortening, respectively, is clearly seen from graphs of strain sweeps (Fig. 10a). The oil batter behaved as a viscoelastic liquid with almost overlapping values of *G*′ and *G*″ at low percent strain. On the other hand, the shortening batter showed viscoelastic solid that could sustain strain of up to 50% before showing permanent deformation (yielding). Among the structured systems, the batter of shellac oleogel came closest to mimicking the batter consistency of shortening reference (albeit with a relatively lower hardness). Additionally, the batter also showed air incorporation equivalent to shortening reference. The functionality of HPMC oleogel as shortening alternative was also reasonably good as seen from a broader LVR compared to the batter of shellac oleogel. However, the HIPE gel batter displayed a much weaker structure with much lower values of moduli and a crossover point at much lower strain.

## Conclusions

In summary, three different approaches of oil structuring were demonstrated using wax crystals, polymer strands, and gelled water droplets as non-TAG-based structurants. While, wax-based oleogels could be prepared using a direct dispersion of shellac wax in liquid oil (at temperatures above the melting point of wax), the HPMC oleogels could only be prepared using an indirect method by first creating a dried porous microstructure followed by oil absorption and subsequent shearing to uniformly distribute polymer strands in the liquid continuous medium. In case of HIPE gels, the oil structuring could only be achieved at a very high volume fraction (*ϕ*_water_ > 0.74) of dispersed water droplets. At such high volume fraction, the network of densely packed gelled droplets was capable of physically trapping thin film of oil continuous phase at the inter-droplet sites. The gelation of water droplet was also necessary to provide physical stability to the system because the emulsion prepared with ungelled water droplets showed phase separation over storage.

The properties for these three types of oleogels were very different from each other. The shellac oleogel showed a fat-mimetic structuring properties due to the reversible melting and crystallization of wax crystals. On the other hand, polymer oleogel was relatively thermostable and maintained a “gel-like” consistency even at high temperatures. The temperature behavior of HIPE gel was similar to the structured water gel showing a reversible gel-sol transformation, albeit at relatively higher critical temperatures. With regards to the thixotropic recovery, although, both the shellac and the HPMC oleogel showed poor structure recovery properties, the recovery of shellac oleogel was significantly better than HPMC oleogel. Shellac oleogel also showed a good tolerability to water incorporation and resulted in the formation of a stable, emulsifier-free w/o emulsions even at water content as high as 60 wt%.

Taking into account the different properties and functionalities of these structured systems, different applications can be envisaged. For example, shellac oleogels would be more suited for preparation of spreads as well as for shortening applications. While, the HPMC oleogels could only be used as water-free shortening alternatives. HIPE gels could be used for creating interesting textures of low-fat products where the mouth feel could be altered by varying the polymer ratios and concentrations in the water phase.

## Abbreviations

Car: carrageenan
Cryo-SEM: cryo-scanning electron microscopy
CSLM: confocal scanning laser microscopy
DSC: differential scanning calorimetry
EC: ethylcellulose
*G*′, *G*″, G*: elastic, viscous and complex modulus, respectively
GL: gelatin
HIPE: high internal phase emulsion
HPMC: hydroxy propyl methyl cellulose
LBG: locust bean gum
LVR: linear viscoelastic region
MAG & DAG: mono- and di-acylglycerols, respectively
MC: methylcellulose
PGPR: polyglycerol polyricinoleate
PLM: polarized light microscopy
REA: ricinelaidic acid
RPO: rapeseed oil
SFO: sunflower oil
TAG: triacylglycerol
*ϕ*_water_: volume fraction of dispersed water phase
w/o: water-in-oil
XG: xanthan gum
3 ITT: 3-interval thixotropy test
12-HSA: 12-hydroxy stearic acid

## Supporting Information

As a service to our authors and readers, this journal provides supporting information supplied by the authors. Such materials are peer reviewed and may be re-organized for online delivery, but are not copy-edited or typeset. Technical support issues arising from supporting information (other than missing files) should be addressed to the authors.

**Figure S1**: (a) DSC profile of shellac showing the melting peaks at 55, 66.4, and 79.4°C and the corresponding crystallization peaks at 69.9, 60.8, and 50.2°C attributed to the chemical composition of shellac and (b) comparative DSC profiles (exothermic heat flow) of shellac dispersion in rapeseed oil at varying concentrations from 0 to 5 wt% shellac, all the samples were cooled at a constant rate of 10°C/min. **Figure S2**: (a and b) Polarized microscopy images of samples prepared at 1 and 10°C/min, respectively.
