# Supplementary material for: Comparative evaluation of structured oil systems: Shellac oleogel, HPMC oleogel, and HIPE gel
Source: Eur J Lipid Sci Technol. 2015 May 5;117(11):1772–81. doi: 10.1002/ejlt.201400553 (PMC4690198; doi:10.1002/ejlt.201400553)
Supplement: Supplementary file 1 [file ejlt0117-1772-sd1.docx]

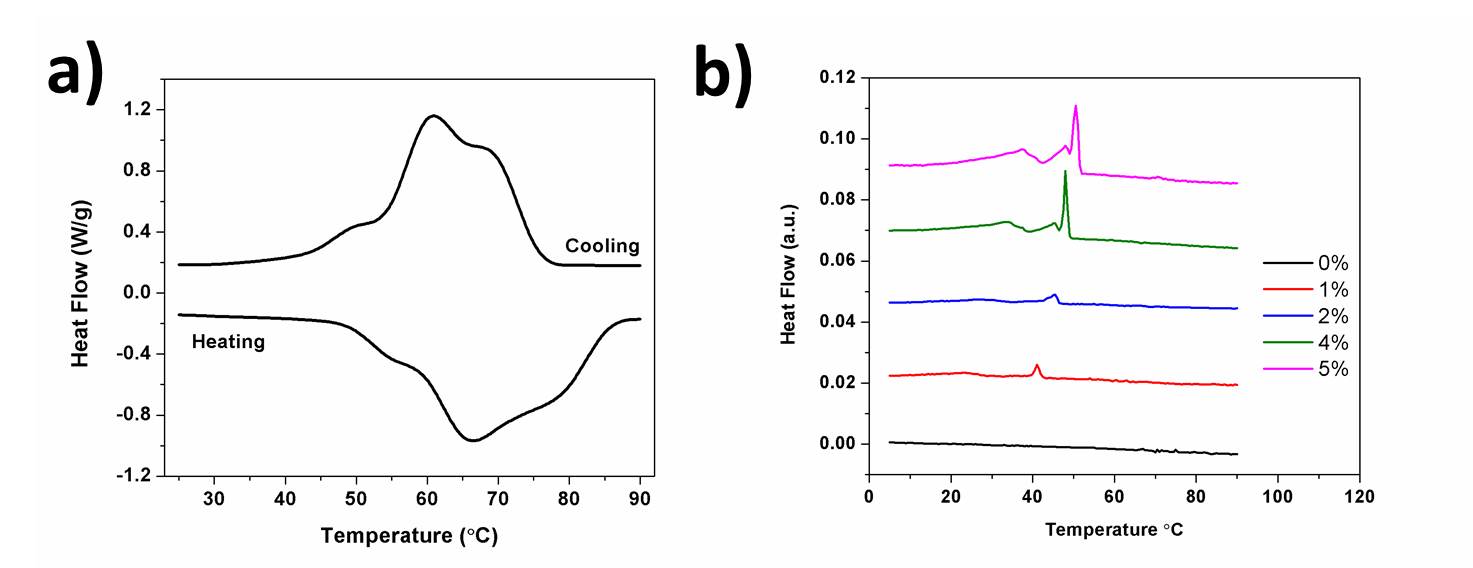


**Fig S1**. a) DSC profile of shellac showing the melting peaks at 55, 66.4 and 79.4 °C and the corresponding crystallization peaks at 69.9, 60.8 and 50.2 °C attributed to the chemical composition of shellac and b) Comparative DSC profiles (exothermic heat flow) of shellac dispersion in rapeseed oil at varying concentrations from 0 to 5 % wt shellac, all the samples were cooled at a constant rate of 10°C/min.


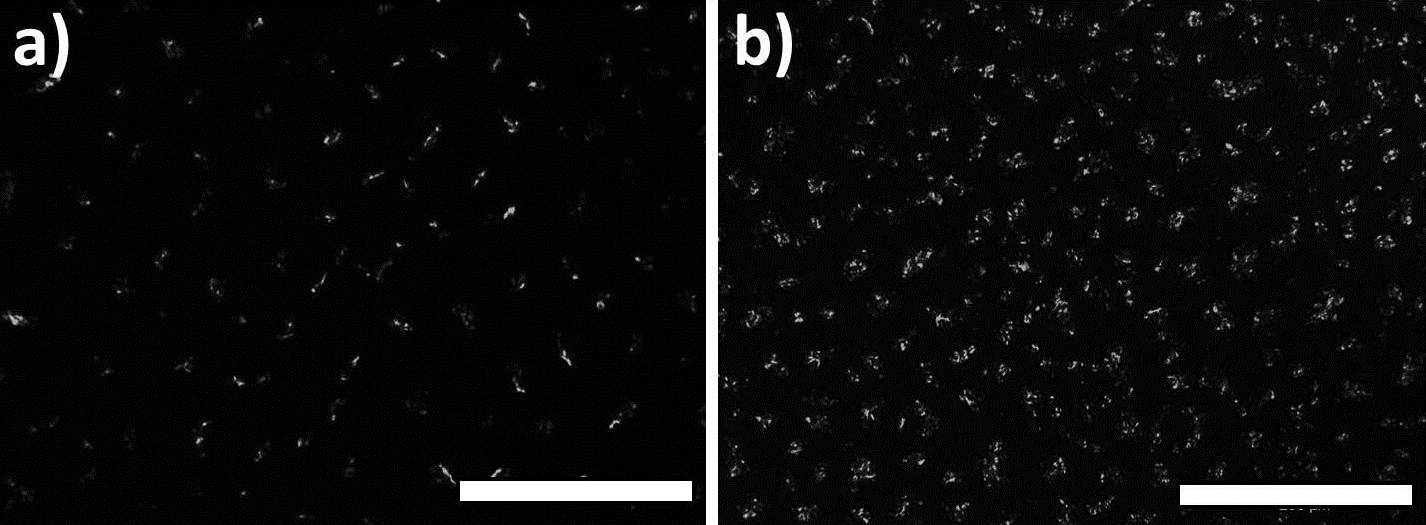


Fig S2. a) & b) Polarized microscopy images of samples prepared at 1 and 10 °C/min respectively.
